# Therapeutic Potentials of Anesthetics

**DOI:** 10.2174/011570159X443578251006094509

**Published:** 2025-10-15

**Authors:** Cheng Zhou, Ji Hu

**Affiliations:** 1 West China Hospital of Sichuan University, Chengdu, China P.R;; 2 School of Life Science and Technology, ShanghaiTech University, Shanghai, China P.R

Modern anesthetics have been widely used in clinical practice for nearly 180 years, which has fundamentally changed the conception of surgery. Anesthetics modulate various brain functions, including but not restricted to consciousness, memory, and sensation. Due to the complexity of the brain, we do not fully understand exactly how anesthetics induce their pharmacological effects, despite the growing knowledge of the molecular targets of anesthetics. Traditionally, anesthetics and perioperative drugs are used to induce a stable physiological state required for surgery, including unconsciousness, amnesia, immobility, muscle relaxation, and preservation of basic life functions (such as heartbeat and respiration). In this sense, anesthetics do not treat any disease *per se*, which may not even belong to the strictly defined concept of “drugs”. This perspective was widely accepted until the last decade. As we all know, the most important progress of psychoactive drugs in the last decade is ketamine, which is a traditional sedative and is now used as a rapid antidepressant [1]. There have been a large number of studies trying to explain how ketamine produces rapid and long-lasting antidepressant effects from molecular to neural circuit levels [2-4], and we look forward to developing novel antidepressants. Therefore, the application of ketamine as an antidepressant also raises the concept that novel psychoactive drugs may be found in traditional anesthetics and perioperative drugs, which were not previously regarded as therapeutic. In the review article by Wang *et al*. [5], the authors summarize the relationship between demyelination and depression, discussing the potential mechanisms by which ketamine may exert its antidepressant effects by repairing myelin damage and offering new insights into the role of myelination in antidepressant mechanisms.

The above concept led to the emergence of a subspecialty of anesthetic pharmacology, termed “Anesthesia Therapy,” which seeks to identify the therapeutic potential of anesthetics. Currently, anesthesia therapy may be one of the fastest-growing research areas [6-10]. Our current thematic issue, “**Therapeutic potentials of anesthetics**”, consists of review articles that focus on research progress in how perioperative drugs are used for neuropsychiatric disorders and/or non-anesthesia purpose treatment. The organ-protective effects of general anesthetics are perhaps the earliest non-anesthetic actions of anesthetics. In the review article by Gabrovic *et al*. [11], the authors summarize the non-anesthetic effects of anesthetics that provide organ-protective benefits against hypoxia-ischemia and its underlying mechanism. This review also provides insights into future clinical trials and applications. In the review article by Pan *et al*. [12], they describe that anesthetic drugs, such as dexmedetomidine, propofol, and stellate ganglion block, are potential therapeutic options for the diagnosis and treatment of sleep disorders. These effects of anesthetics on sleep can help improve sleep quality and reduce the incidence of sleep disorders, thereby guiding clinical medication and improving the prognosis of patients. However, clinical evidence in this area are still limited, and there is significant heterogeneity among different studies. Large-scale clinical trials are further needed to enhance the certainty of these conclusions. The review article by Song *et al*. [13] explores the developmental neuroplasticity during the critical periods, and the dual roles of general anesthetics’ action on neural circuits, including the current understandings of their neurotoxic and neuroprotective effects based on the alterations of neuroplasticity.

Furthermore, this review also highlights the emerging therapeutic potential of general anesthetics for neurological disorders with impaired neuroplasticity as the core pathological mechanisms, with a particular emphasis on the neurodevelopmental disorders and autoimmune neurological disorders. It proposes directions for future research to unlock their full clinical utility. In the review article by Leng *et al*. [14], the authors discuss the effects and mechanisms of anesthetics and/or analgesics on intraoperative neurophysiological monitoring (IONM), with a focus on evoked potentials. It provides critical guidance for selecting anesthetic protocols to improve monitoring accuracy and surgical outcomes, while also offering insights into the neural mechanisms of anesthetic actions. Future research will focus on standardizing intraoperative neurophysiological monitoring criteria and leveraging technological advances to refine personalized monitoring strategies and enhance intraoperative safety. Besides the thematic issue, a previously published review article by Hu *et al*. [15] also summarizes the therapeutic potential of dexmedetomidine in neuropsychiatric disorders, such as Alzheimer’s disease, Parkinson’s disease, and anxiety, emphasizing its neuroprotective, anti-inflammatory, and anti-apoptotic effects beyond sedation. Its sublingual form, BXCL501, is an FDA-approved drug for agitation, demonstrating clinical relevance. Future studies should clarify its mechanisms, optimize dosing, and advance clinical applications.

In conclusion, increasing studies highlight the potential of anesthetics as non-anesthetic therapy for varied neuropsychiatric disorders, including but not limited to brain ischemia, depression and anxiety, sleep disorders, *etc*. As researchers continue to advance, it is necessary to combine multidisciplinary studies, especially clinical trials, to find and/or confirm the therapeutic potential of anesthetics. This endeavor holds great promise for the development of emerging therapy strategies and perioperative drugs, which also helps us understand our brain.

As the guest editors, we would like to express our gratitude to the authors who contributed to this special issue, who share their various aspects of neuropharmacology related to “Therapeutic potentials of anesthetics”. It is clear that such review articles represent only a small part of our understanding of the potential applications of anesthetics as “therapeutic drugs.” Therefore, we hope this special issue serves as a catalyst for further investigation and innovation in this promising field.
